# When undernutrition meets overweight: prevalence and determinants of double burden of malnutrition among women-child pairs in Burkina Faso from the 2021 Demographic and Health Survey

**DOI:** 10.1186/s12889-026-27603-x

**Published:** 2026-05-02

**Authors:** Baperman Abdel Aziz Siri, Diana Sagastume, Grace Marie Ku, Franck Garanet, Hervé Hien, Lenka Beňová

**Affiliations:** 1 Ministry of Health, Ouagadougou, Burkina Faso; 2 Institute of Tropical Medicine (ITM), Antwerp, Belgium; 3 Institut de recherche en science de la santé (IRSS), Centre national de recherche scientifique et technologique (CNRST), Ouagadougou, Burkina Faso

**Keywords:** Double burden of malnutrition, Anemia, Overweight/obesity, Household, Burkina Faso

## Abstract

**Background:**

Burkina Faso is experiencing a rapid nutritional transition, yet evidence on the double burden of malnutrition (DBM) at household and individual levels remains limited. This study aimed to estimate the national prevalence of DBMs and identify associated factors with a particular focus on the most common type of DBM.

**Methods:**

We used data from the 2021 Burkina Faso Demographic Health Survey, a nationally representative cross–sectional survey. The analysis included 4119 households with 5146 women of reproductive age (15–49 years) and 4114 mother-child pair. Eleven (11) potential combinations of maternal and child nutritional status indicators, defined according to WHO standards (anemia, stunting, wasting, underweight, and overweight/obesity) were assessed. Weighted descriptive and bivariate analyses were conducted to identify the most prevalent double burden of malnutrition (DBM) combination, which was selected as the primary outcome. Factors associated with this outcome were examined using multivariate logistic regression with four nested models incorporating household, maternal, and child level characteristics.

**Results:**

Overall, 72% of children were affected by anemia, 23% by stunting, and 17% were in underweight condition. Among women, 53% were living with anemia, 17.9% with underweight, and 7.6% with obesity. The predominant DBM form was children with anemia combined with overweight/obesity in women, affecting 14.3% of households. The individual prevalence of anemia and overweight/obesity in women was 9.47%, while stunting paired with overweight in children affected 3.6% of households. Multivariate analysis identified several independent of the predominant form of DBM: region (aOR = 1.94, 95% CI = 1.09–3.45 for Sahel region vs. Boucle du Mouhoun), household wealth (aOR = 3.1, 95% CI = 1.74–5.53 for richest vs. poorest), place of residence (aOR = 0.6, 95% CI = 0.45–0.79 for rural vs. urban), women’s age (aOR = 6.48, 95% CI = 2.53–16.61 for 35–39 years compared to 15–19), and women’s occupation (aOR = 2.71, 95% CI = 1.5–4.87 for professional manager vs. not working).

**Conclusion:**

The most common DBM, child with anemia and maternal overweight/obese women affect more than one in seven households in Burkina Faso, with a higher risk in urban and wealthier settings. Integrated “double duty” interventions are urgently needed to address both mother with overweight/obesity and child with anemia while addressing the structural drivers of nutrition transition and broader social determinants of health.

**Supplementary Information:**

The online version contains supplementary material available at https://doi.org/10.1186/s12889-026-27603-x.

## What is already known on this topic

In low and middle-income countries, undernutrition and overnutrition coexist, reflecting ongoing nutritional transitions. Prior evidence highlights strong socioeconomic gradients: anemia and overweight/obesity often coexist in urban and wealthier households. However, most existing studies have examined individuals in isolation rather than considering how different forms of malnutrition cluster within families. Research on the coexistence mother with overweight/obesity and child with anemia within the same household remains very limited, particularly in West Africa and Burkina Faso.

## What this study adds

This study provides the first national assessment of the Double Burden Malnutrition (DBM) in Burkina Faso using the 2021 Demographic and Health Survey (DHS). It adopts an exploratory approach systematically examining eleven combinations of under and overnutrition at both the individual and household levels, allowing the identification of the most relevant forms of DBM. The findings show that the most common DBM configuration is the coexistence of child with anemia and mother with overweight/obesity within the same household, with clear socioeconomic and regional disparities shaping this pattern.

## How this study might affect research, policy and practice

The findings highlight the urgent need for household-level, double-duty actions that address child micronutrient deficiencies and maternal overweight/obesity simultaneously, especially in urban and wealthier families, where this dual burden is most pronounced. For researchers, the study offers a robust, scalable framework for analyzing how multiple forms of malnutrition intersect within households, encouraging deeper investigation into shared dietary, behavioural and environmental pathways affecting all family members.

## Introduction

Malnutrition encompasses both undernutrition, including stunting, wasting, and underweight, micronutrient deficiencies, and overnutrition, represented by overweight and obesity. It remains a pressing global public health challenge. In 2022, more than 2.5 billion adults worldwide were overweight or obese, while 390 million adults were underweight [1]. Among children under five years of age, the situation is even more critical: 149 million have stunted, 45 million have wasted, and 37 million are overweight or obese, with projections suggesting a 254.8% increase in overweight and obesity by 2050 [2]. These figures highlight the persistent and complex nature of the burden of malnutrition worldwide [3].

Historically, different forms of malnutrition have been studied and addressed as distinct public health challenges. However, growing evidence shows that multiple forms of malnutrition can coexist [4–6]. The double burden of malnutrition (DBM) refers to the coexistence of undernutrition along with overweight, obesity, or diet–related noncommunicable diseases (NCDs) within individuals, different members of the same households, or populations and across the life course [7], reflecting distinct but interconnected biological and social pathways. At the household level, this may manifest, for example, as an overweight or obese mother living with an undernourished child, whether through wasting, stunting or specific micronutrient deficiencies. At the individual level, this may present as overweight/obesity combined with stunting or micronutrient deficiency within the same person.

The global burden of DBM has become increasingly critical, particularly in low- and middle-income countries (LMICs), where persistent undernutrition among children coexists with a rapidly rising prevalence of overweight and obesity in adults. This dual burden reflects nutritional and epidemiological transition driven by urbanization, economic development and shifts in dietary practices [5, 8]. Nowhere is this convergence more apparent than in Sub Saharan Africa (SSA), which accounts for over 30% of the world’s children with stunting while simultaneously experiencing a growing epidemic of adult obesity [8, 9]. Within this changing landscape, living conditions, and health risks are shared, shaping the nutritional status of its members [4].

To date, research on the DBM has largely focused on mother child pairs, most commonly characterised by the coexistence of child with stunting and mother with overweight or obesity [10, 11]. While this dyad provides important insights, it captures only part of broader more complex phenomenon. DBM reflects the interaction of multiples vulnerabilities operating across interconnected levels. Ath the household level, some factors such as higher wealth quintiles, lager household size, food insecurity, urban residence and inadequate sanitation have been associated with increased risk [10, 11]. Maternal characteristics, including older age, high parity and intermediate levels of education further shape this risk profile, while child level factors, such as age 6–23 months, suboptimal infant and young feeding practices, and recurrent infections compound these vulnerabilities [12–14]. Together, these elements highlight the multilevel and interdependent nature of DBM.

Burkina Faso exemplifies these overlapping nutritional challenges. Although child stunting has declined from 35% to 25% between 2010 and 2021 [15], it remains a major public health concern. At the same time, the prevalence of overweight among women of reproductive age has increased from 8.1% to 13.7%, and obesity from 3.1% to 6.2%, accompanied by a rising burden of non-communicable disease [16]. Thes parallel trends signal the emergence of DBM as a critical and evolving public health issue.

Despite growing recognition of DBM, important gaps persist. Evidence remains limited for less commonly examined forms, such as the coexistence of maternal overweight or obesity alongside child with anemia, and few studies have simultaneously captured DBM at both individual and intrahousehold levels. Most existing studies focused on a narrow set of indicators, thereby providing a partial view of the DBM scope. In SSA, available evidence remains sparse, although a multicuntry analysis reported DBM prevalence ranging from 3,7% to 17% between 2008 and 2017, with marked heterogeneity across stings undergoing rapid nutritional transition [10]. Notably, prevalence estimates up 38% when broader definitions, including maternal overweight or obesity combined with child anemia, were considered [11], underscoring the importance of capturing the full spectrum of DBM.

In Burkina Faso, existing studies have begun to explore DBM [17, 18], yet their scope remains limited. To our knowledge, no study has used nationally representative data to jointly estimate DBM prevalence at both household and individual levels while examining the combined influence of sociodemographic and contextual factors. Addressing this gap is particularly important in a setting marked by rapid nutritional transition [19, 20], increased urbanization, and persistent inequalities in access to healthcare [21]. Considering both intrahousehold and individual dimensions allows for more comprehensive understanding of DBM, capturing shared exposures alongside individual susceptibilities. This perspective is further supported by evidence highlighting the central role of maternal status in shaping child growth trajectories and long-term health outcomes [6, 8].

Building on this evidence, we posit that DBM arises from interplay of factors operating across maternal, household and contextual and child levels, structured along a distal to proximal pathway [10, 17, 22, 23]. This conceptual perspective guided the selection of variables included in the analysis. Elucidating these multilevel mechanisms is essential to better understand how undernutrition and overnutrition can coexist within the same individuals and households. Understanding the scope of DBM scope and its underlying factors is critical for informing context specific integrated nutrition policies.

Using nationally representative data from 2021, this study aims to estimate the prevalence of DBM at both household and individual levels in Burkina Faso and to identify its associated factors across maternal, household and child characteristics with particular attention to its most common forms.

## Methods

### Study design, data source, and sampling design

We conducted a cross-sectional study, secondarily analysing data from the Demographic and Health Survey (DHS) 2021. The DHS is nationally representative and provide estimates at national, regional and urban-rural level. It includes information on households, women of reproductive age (15–49 years), and children under 5 years of age.

The survey employed a stratified two-stage cluster sampling design. In the first stage enumeration area (cluster) were selected from the 2019 population census using probability proportional to size. In the second stage, a random of household was selected within each cluster. Each of the 13 administrative regions was stratified into urban and rural sampling strata (26 in total). A total of 15,600 household was planned for inclusion (5,070 urban; 10,530 rural), distributed across 600 clusters (195 urban, 405 rural) selected with probability proportional to size form the 2019 census [24]. Anthropometric measurements and biomarker data were collected only in a subsample (one out of two households); therefore, not all households contributed to the analysis. A detail description of the survey methodology, including sampling procedures has been reported elsewhere [15].

### Study site

Burkina Faso, a West African country with 13 administrative regions, had a population of 20,505,155 in 2019, with an annual growth rate of 2.94%. The average household size was 5.2 persons, and 26.1% of population resided in urban areas. Women of childbearing age (15–49 years) numbered 4,949,953 (24.1% of the total population and 46.7% of females), while children under five years numbered 3,319,064 (16.2% of the total population [24].

### Study population

#### Sample construction

Data from the household, women, and children files were merged using standard identifiers to reconstruct the hierarchical household-women-child structure. The analysis focused on mother child dyads residing in the same households and individual nutritional double burden.

#### Inclusion criteria

Children aged 6–59 months with an identified mother were included. Valid height for age Z score (HAZ) measurement was required and defined as between − 6 and + 6 standards deviation (SD).

Women aged 15–49 years were included. Pregnant women and those within six months postpartum were excluded to reduce bias related to physiological weight change. Women were required having a valid body mass index (BMI), defined between 12 and 60 kg/m2.

#### Sample size and analytic samples

First, an individual level sample was used to assess intra-individual DBM, including all eligible women and or children with available valid data. Analyses were restricted to complete cases, and no imputation was performed. Sample size varied across indicators depending on data availability, with a maximum of 5146 women and 5105 children included.

Second, a mother-child dyadic sample was used to assess inter-individual (household level) DBM. It included matched pairs with complete data on maternal BMI and child anthropometry, yielding 4,114mother-child dyads.

### Measurement of variables

#### Data collection tools and procedures

This study used secondary data from a nationally representative DHS survey. The original data were gathered through carefully trained interviewers using standardized household, women’s, and biomarker questionnaires, along with high-quality anthropometric and hemoglobin measurements collected under strict quality control procedures( 15).

#### Outcome variable

The DBM was defined as the coexistence of overnutrition and undernutrition, either within the same individual (women or child) or between the same mother and child within the same household in order to capture situations of nutritional discordance. Based on this definition, 11 possible combinations of DBM were identified in the available data and are presented in Table 1. These DBM combinations were based on four child malnutrition indicators (stunting, wasting, underweight, anemia, and overweight) and four indicators in women (underweight, short stature and anemia, and overweight). Nutritional indicators were defined according to WHO standards and cut-offs [25–27].

**Table 1:** Variables of construction of DBMs (typologies)

		Undernutrition						
		Children 6–59 months				Women 15–49 years old		
		Stunting (Z score Heigh-for-Age <-2 SD)	Wasting (Z score Weight-for-Height <-2 SD)	Underweight (Z score Weight-for-Age <-2 SD)	Anemia (Hb < 11 g/dl)	Anemia (Hb < 12 g/dl)	Undernutrition BMI < 18.5 kg/m^2^)	Short stature (Height < 145 m)
Overnutrition	Overnutrition in children (Z score Weight-for-Height ≥ + 2 SD)	X			X	X	X	X
	Overweight/obesity in women (BMI ≥ 25Kg/m^2^)	X	X	X	X	X		X

#### Definition of undernutrition

In women, Underweight was defined as a body mass index (BMI) < 18.5 kg/m².

In children under five years of age: Stunting was defined as Height-for-age z-score (HAZ) < -2 standard deviations (SD), Wasting as a Weight-for-height z-score (WHZ) < -2 SD and Underweight as Weight-for-age z-score (WAZ) < -2 SD.

Short stature (study-specific) was defined as height < 145 cm in the study population.

All thresholds above (BMI < 18.5 kg/m², HAZ/WHZ/WAZ < -2 SD, and height < 145 cm) were collectively classified as indicators of undernutrition.

#### Definition of overnutrition

In this study, overnutrition was defined as a body mass index (BMI) ≥ 25 kg/m² in women and a weight-for-height z-score ≥ + 2 standard deviations (SD) in children under five years of age.

#### Anaemia

In women aged 15–49, anaemia was defined as a Haemoglobin level < 12 g/dl in. Anaemia in children was defined as a haemoglobin level < 11 g/dl.

#### Other variables

**Fig. 1:**
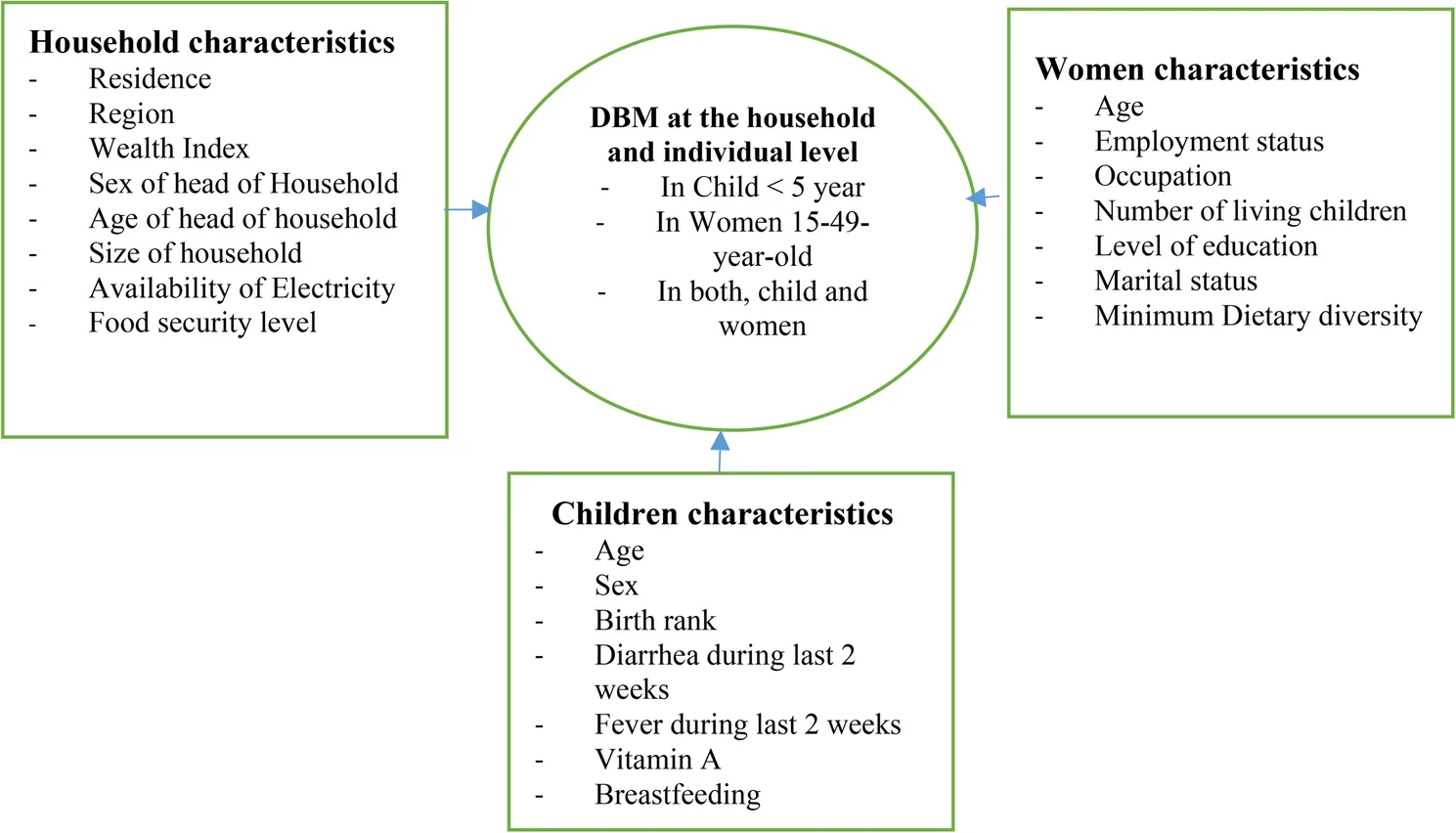
Conceptual framework of DBM associated factors in our study

Figure 1 illustrates the potential factors associated with DBM investigated in this study based existing evidence [10, 12, 13, 17, 22, 23, 28] and theorical relevance [29], as well as the availability of variables from the Burkina Faso 2021 DHS dataset. The choice of explanatory variables was guided by our study hypothesis. To empirically assess this hypothesis, we identified and grouped relevant variables from the Burkina Faso DHS 2021 dataset into these four domains.

#### Household characteristics

The contextual factors included geographic region (Boucle du Mouhoun, Cascades, Centre, Centre-Est, Centre-Nord, Centre-Sud, Est, Hauts-Bassins, Nord, Plateau-Central, Sahel, Sud-Ouest) and place of residence (urban/rural); availability of electricity (yes/no); age of the head of household (< 25, 25–34, 35–44, 45–54, 55–64, ≥ 65 years); food insecurity level (absent, Mild, moderate, severe); household size (1–2, 3–4, 5–6, and ≥ 7 member); wealth index (poorest, poorer, middle, richer, richest); and sex of the head of household (male/female).

#### Women-related factors

The characteristics of the women included the following: employment status as currently working (yes/no), educational level (no formal education, primary, secondary, or tertiary), marital status (never married, married, living with partner, widowed, Separated, no longer living with partner), age group (15–19, 20–24, 25–29, 30–34, 35–39, 40–44, 45–49 years), occupation type (unemployed, professional/technical, administrative, sales, agriculture, self-employed/salaried, domestic, other), number of living children (0, 1, 2, 3–4, 5–6, ≥ 7), and minimum dietary diversity (MDD) (< 5 food groups, ≥ 5 food groups) [15].

#### Child-related factors

The child-level variables included age in months (6–11, 12–24, 24–36, 36–59), sex (male/female), birth rank (1, 2–3, ≥ 4), recent illnesses such as diarrhea or fever episodes in the last two weeks (yes/no), supplementation with vitamin A and deworming treatment in the past six months (yes/no), and breastfeeding (ever breastfed, not currently breastfeeding, and still breastfeeding).

### Data analysis

All analyses were conducted via R 3.14 software and accounted for complex survey design of the DHS data, including clustering at the primary sampling unit, stratification and sampling weights to ensure valid variance estimation.

Descriptive statistics (means and standard deviations or medians and interquartile ranges for continuous variables and frequencies and percentages for categorical variables) were computed to summarize the distribution of the characteristics of the sample. The different types and combinations of DBM at the household and individual levels were categorized as binary outcomes independently: 1 if the phenomenon exists and 0 otherwise.

The prevalence of the different combinations of DBMs (at the individual and household levels) was calculated by dividing the number of individuals exposed to each specific combination of DBMs (n) by the total number of eligible individuals in the sample (N), with a corresponding 95% confidence interval. The combination with the highest prevalence was selected for further analysis. For this purpose, both bivariate and multivariable logistic regression analyses were performed.

Bivariate analyses were conducted via survey-weighted regression models to examine associations between the most prevalent DBM combination and explanatory variables, as illustrated in Fig. 1.

Variables with a p-value < 0.20 in the bivariate analysis were retained for inclusion in multivariate models. To ensure the reliability of the multivariate models, multicollinearity was assessed via variance inflation factors (VIFs) with a value threshold of 5.

A hierarchical modelling approach was used, in which variables were introduced in a sequentially from distal to proximal determinants, as recommend in epidemiological analyses [30] and informed by the UNICEF conceptual model of malnutrition [29]. This strategy was chosen for two reasons. First, DHS data are inherently hierarchical, with children nested within mothers, themselves nested within households. Ignoring this clustering underestimates standard errors and inflates Type I error [31]. Second, block wise entry quantifies each level’s contribution to DBM risk.

We then adopted a stepwise hierarchical modelling approach, where explanatory variables were introduced progressively across four nested models:

- Model I: adjusted for women’s sociodemographic characteristics (age, education level, marital status, and occupation).
- Model II: addition of household-level characteristics (wealth index, access to electricity, region, place of residence, age of household head, and food insecurity status).
- Model III: incorporation of child-related variables (child’s age, breastfeeding status, and recent fever).
- Model IV (full model): further included women’s dietary diversity, considered as a proximal determinant of nutritional status, in addition to the previously included covariates.

Survey-weighted logistic regression models were fitted using the svyglm function with a binomial family, and results were reported as adjusted odds ratios(aORs) with 95% confidence intervals. To assess the incremental contribution of each block of variables, nested models were compared using design adjusted Wald tests, with a p-value < 0.05 indicating improved model fit. Model performance was further evaluated using pseudo-R² (McFadden, Cox & Snell, and Nagelkerke), with particular emphasis on Nagelkerke’s index for interpretation.

### Sensitivity analysis

To assess the robustness of our findings to potential within mother correlation arising from 762 mothers (22.7%) contributing multiple children, we conducted a sensitivity analysis by randomly selecting on child per mother using a reproductible random seed (*n* = 3,352). Survey weighted logistic regression models were re-estimated with identical specifications, and results were compared with the main analysis (*n* = 4,114) regarding direction, magnitude, and statistical significance of association.

### Ethical approval

The DHS receives government permission, uses informed consent and assures respondents of confidentiality. We did not require ethics approval for this study since it involved secondary analysis of anonymized data.

## Results

### Study sample and population characteristics

**Fig. 2:**
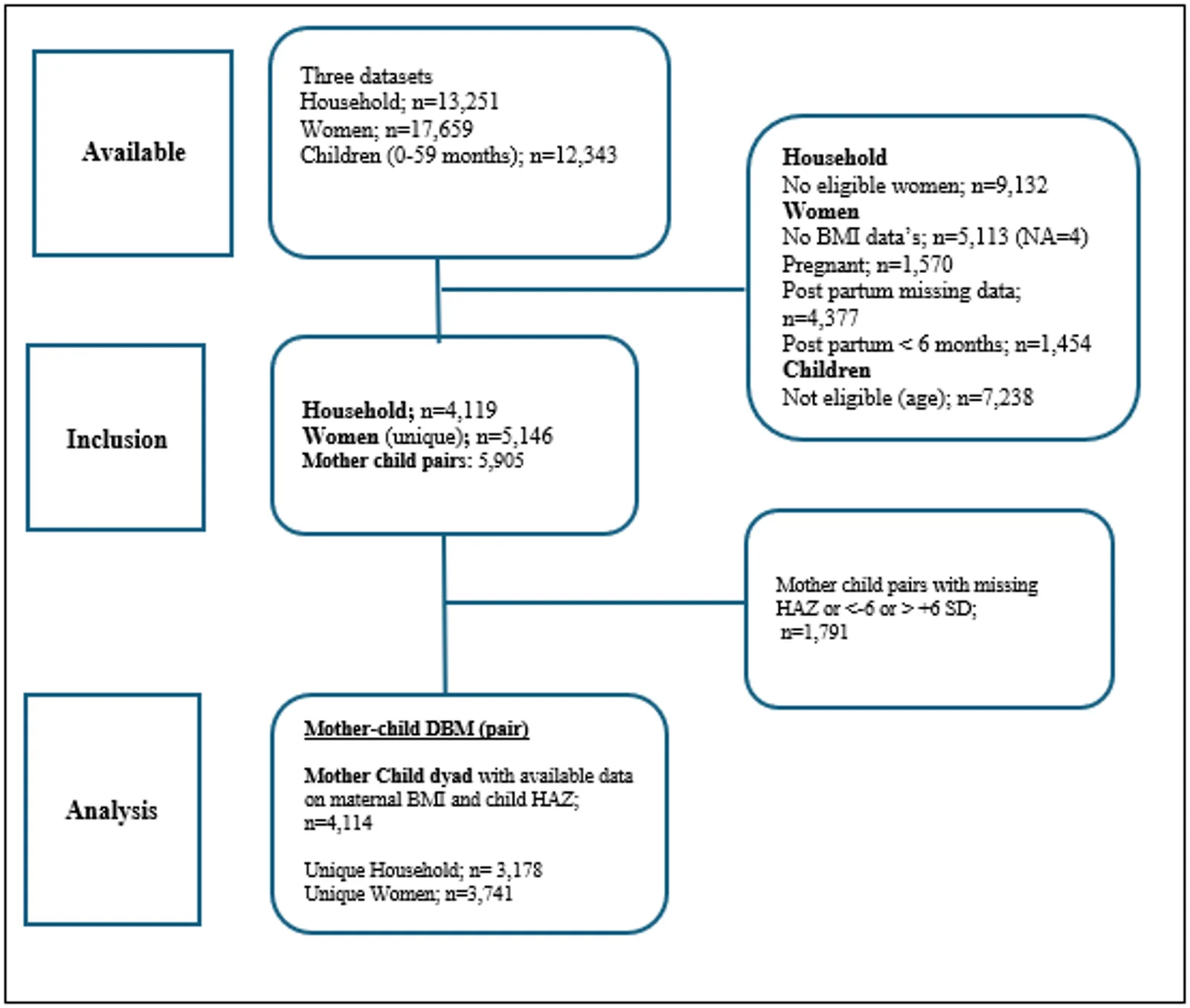
Flow chart of study sample selection and analytic populations in the 2021 Burkina Faso DHS

The sample selection process is presented in the Fig. 2. A total of 4,119 households, 5,146 women and 4114 mother-child dyads were included. The individual level sample included all eligible participants with available data. The mother child dyadic sample, restricted to pairs with complete and valid maternal BMI and child anthropometric data (HAZ), comprised 4,114 dyads, corresponding to 3, 178 households and 3,741 women (762 have ≥ 2 children). The difference between the two samples reflects data availability and matching requirements.

The characteristic of the study sample is described on Table 2. The median household size was 7 members (IQR: 5–9). The majority (58.2%) of the households experienced some degree of food insecurity, with 35% classified as severely food insecure. Among women of reproductive age, the predominant age groups were 30–34 years (18.3%), 35–39 years (17.9%), and 25–29 years (17.5%), with a median age of 33 years. The mean number of living children was 3, with 34.5% of women having 3–4 children and 22.3% having 5–6 children. Dietary diversity was limited, as 65.5% of women consumed fewer than five food groups.

Children had a median age of 30 months, with 40.6% under 24 months having the largest age group of 36–59 months (30.7%). The majority of children had been breastfed at the time of the survey, with around two-thirds still breastfeeding, one quarter having stopped, and only a small proportion never breastfed. (Table 2).

**Table 2:** Characteristics of the study sample

Variable	*n* (unweighted)	Weighted Frequency %	95%CI Frequency %
Context related			
Region			
Centre	469	19.1	(17.4–20.9)
Hauts-Bassins	441	10.4	(9.4–11.4)
Centre-Est	385	10.2	(9.3–11.2)
Centre-Ouest	359	10.1	(9.1–11.2)
Boucle du Mouhoun	337	9.4	(8.6–10.3)
Centre-Nord	280	8.6	(7.4–9.8)
Est	214	6.3	(5.4–7.2)
Nord	334	6	(5.2–6.8)
Plateau-Central	333	6	(5.4–6.6)
Centre-Sud	271	4.6	(4.1-5.0)
Sud-Ouest	332	3.7	(3.1–4.3)
Cascades	233	3.3	(2.8–3.8)
Sahel	131	2.1	(1.4–2.8)
Place of residence			
rural	2727	69	(67.2–70.8)
urban	1392	31	(29.2–32.8)
Household related			
Electricity			
no electricity	3261	79.5	(76.9–82.0)
yes electricity	858	20.5	(18.0-23.1)
Age of the head of household			
< 25	118	2.8	(2.2–3.5)
35–44	1220	29.8	(28.2–31.5)
45–54	971	23.3	(21.9–24.8)
25–34	801	19.8	(18.3–21.4)
55–64	552	13.1	(11.8–14.3)
65+	457	11	(9.9–12.2)
Median(Q1-Q3) = 44(35–54)			
Food insecurity level			
Absent/Mild	1693	41.9	(39.5–44.3)
severe	1424	35	(32.7–37.2)
moderate	976	23.2	(21.5–24.8)
Size of the household (members)			
> 7	2066	49.9	(47.9–51.9)
5–6	1140	27.8	(26.1–29.5)
3–4	829	20.1	(18.6–21.5)
1–2	84	2.2	(1.6–2.7)
Median(Q1-Q3) = 7 (5–9)			
Wealth Index			
Richest	877	22.5	(20.4–24.6)
Richer	896	20.5	(18.6–22.5)
Middle	877	20.2	(18.6–21.7)
Poorest	722	18.8	(17.0–20.6)
Poorer	747	18.1	(16.6–19.5)
Sex of the head of household			
Male	3552	86.7	(85.3–88.0)
female	567	13.3	(12.0–14.7)
Women of reproductive age			
Currently working			
Yes working	3498	67	(64.8–69.2)
Not working	1648	33	(30.8–35.2)
Education level			
No formal education	3693	72.1	(70.2–74.0)
secondary	773	14.4	(13.1–15.7)
primary	616	11.9	(10.7–13.1)
tertiary	64	1.6	(1.1–2.1)
Mother’s marital status			
married	3474	66.8	(64.5–69.0)
Living with a partner	1229	25	(22.8–27.2)
Never	167	3	(2.5–3.6)
widowed	147	2.7	(2.2–3.3)
No longer living partner	104	1.9	(1.5–2.3)
Divorced	25	0.5	(0.2–0.9)
Mother’s age group			
30–34	926	18.3	(17.0–19.5)
35–39	927	17.9	(16.7–19.2)
25–29	895	17.5	(16.2–18.8)
40–44	812	15.5	(14.4–16.6)
20–24	762	14.5	(13.4–15.6)
45–49	629	12.3	(11.2–13.4)
15–19	195	4	(3.4–4.6)
Median (Q1-Q3) = 33 (26–40)			
Occupation			
Agricultural employee	1888	36	(33.5–38.5)
Not working	1452	29.4	(27.3–31.6)
sales	1188	22.2	(20.5–23.9)
skilled manual	172	3.5	(2.9–4.2)
Professional/technical/managerial	163	3.3	(2.7–4.0)
Service	89	1.8	(1.4–2.2)
Domestic employee	73	1.6	(1.1–2.1)
Unskilled manual	84	1.6	(1.2–2.0)
Clerical	20	0.5	(0.3–0.7)
Number of living children			
3–4	1776	34.5	(33.0–35.9)
5–6	1174	22.3	(21.0–23.7)
1	936	18.5	(17.2–19.9)
2	841	16.5	(15.4–17.6)
≥ 7	398	7.8	(6.9–8.6)
0	21	0.4	(0.2–0.6)
Median (Q1-Q3) = 3 (2–5)			
Dietary diversity			
< 5 food groups	326	65.51	(60.61–70.41)
≥ 5 food groups	162	34.48	(29.58–39.38)
Children related			
Child’s age in months			
6–12	587	14.01	(12.86–15.17)
12–24	1096	26.60	(24.99–28.23)
24–36	800	19.72	(18.28–21.16)
36–59	1631	39.65	(38.09–41.22)
Median (Q1-Q3) = 30(17–44)			
Child Sex			
Male	2121	51.35	(49.69–53.01)
Female	1993	48.64	(46.99–50.31)
Birth’s Rank			
1	842	20.88	(19.46–22.31)
2	720	17.52	(16.39–18.67)
3	709	17.21	(15.98–18.45)
≥ 4	1843	44.36	(42.41–46.32)
Diarrhea in the last two weeks			
Yes	706	17.39	(15.97–18.81)
No	3404	82.60	(81.19–84.03)
Fever in the last two weeks			
Yes	1051	25.92	(24.13–27.73)
No	3059	74.07	(72.27–75.87)
Vitamin A during the last 6 months			
Yes	1192	47.34	(44.26–50.43)
No	1168	52.65	(49.57–55.74)
Breastfeeding			
Ever breastfed, not currently breastfeeding	575	24.89	(22.76–27.03)
Still breastfeeding	1537	63.66	(61.41–65.91)
Never breastfed	282	11.43	(9.9–12.98)

### Prevalence of malnutrition among women of reproductive age and children

Among the children under five years of age, 72% (95% CI: 70.7–74.5) had anemia, 23.3% had stunting (95% CI: 21.4–25.1), 17.7% (95% CI: 16.3–19.1) were underweight, and 10.6% (95% CI: 9.3–11.8) had wasting (95% CI: 9.3–11.8). Childhood overweight was rare, at only 1% (95%: 0.6–1.4%). Among women of reproductive age, 53.9% (95% CI: 52.2–55.6) had anemia, 9.8% (95% CI: 8.7–10.8) were underweight, 17.9% (95% CI: 16.6–19.3) were overweight, and 7.6% (95% CI: 6.7–8.5) were obese (Table 3).

**Table 3:** Distribution of the prevalence of independent forms of malnutrition in children under five years of age and women 15–49 years of age

	*N*/*n* Unweighted	Prevalence % 95 CI Weighted
Children		
Stunting	4114/930	23.28(21.44–25.12)
Underweight	4111/699	17.71(16.31–19.10)
Wasting	4103/406	10.55(9.33–11.77)
Overnutrition	4103/42	1.03(0.6–1.44)
Anemia	4095/2972	72.59(70.67–74.50)
Women		
Underweight	5146/471	9.80(8.7–10.8)
Overweight	5146/956	17.90(16.6–19.3)
Obesity	5146/389	7.60(6.7–8.5)
Anemia	5146/2756	53.90(52.2–55.6)
Height < 145 cm	5133/13	0.30(0.1–0.5)

### Prevalence of DBM at individual and household levels

As shown in Table 4, the overall prevalence of DBMs, which combines all forms across the household and individual levels, was 30%. At the household level, 20% of households experienced at least one form of DBM. The most common DBM combination at the household level was the coexistence of anemia and maternal overweight/obesity, at 14.3% (95% CI: 12.9–15.8). The coexistence of child stunting and maternal overweight was the second most prevalent household-level combination, estimated at 3.6% (95% CI: 2.9–4.3), followed by child underweight and maternal overweight (2.4%, 95% CI: 1.8–2.9). At the individual level, the most common DBM form was coexisting anemia and overweight in women, with a prevalence of 9.5% (95% CI: 8.3–10.7).

**Table 4:** Prevalence of the 11 combinations of DBMs at the individual and household levels

Combination of DBMs	Unweighted Frequency *N*	*n*	Weighted Prevalence (%)	95%CI
Household				
A child with stunting and a woman with overweight (DBM1)	4114	150	3.60	(2.89–4.30)
A child with wasting and a woman with overweight/obesity (DBM2)	4103	64	1.72	(1.22–2.22)
A child with Underweight and a woman with overweight/obesity (DBM3)	4111	97	2.36	(1.84–2.89)
A child with anemia and a woman with overweight/obesity (DBM4)	4095	610	14.32	(12.85–15.77)
A child with overweight/obesity and a woman with short stature (DBM5)	4103	0	-	-
A child with overweight/obesity and a woman with underweight (DBM6)	4103	3	0.08	(0.02–0.19)
A child with overweight/obesity and a woman with anemia (DBM7)	4088	24	0.65	(0.32–0.97)
Estimated overall prevalence			20	(18–22)
Individual				
A woman with anemia and overweight/obesity (DBM8)	5133	387	9.47	(8.27–10.65)
A woman with short stature and overweight/obesity (DBM9)	5146	5	0.09	(0.015–0.196)
A child with stunting and overnutrition (DBM10)	4103	26	0.57	(0.28–0.85)
A child with anemia and overnutrition (DBM11)	4084	30	0.78	(0.39–1.15)
Estimated overall prevalence			9.8	(8.5–11)
Estimated prevalence (household and individual level)			30	(27–33)

### Factors associated with the DBM combination in children with anemia and overweight women

This combination was the most prevalent among the 11 DBMs type assessed.

Table 5 presents the results of the bivariate and multivariate logistic regression between household, women and child characteristics associated with the DBM combination of children with anemia and maternal overweight/obesity (complement in Annex 2).

**Table 5:** Bivariate and multivariate analysis of factors associated (household) to the DBM combination of child with anemia and women with overweight/obesity (DBM4)

	*N*/*n*	Proportion (weighted)	OR95%CI	*p*-value Wald	Model II_aOR (95% CI)^1^	*p* value
Household						
Region						
Boucle du Mouhoun	392/55	14.1	0.16 (0.10–0.26)	*p* < 0.0001	Ref	
Cascades	126/27	21.6	1.68 (0.86–3.31)		0.91 (0.43–1.9)	0.801
Centre	616/166	26.9	2.25 (1.34–3.76)		0.79 (0.47–1.34)	0.385
Centre-Est	452/45	10	0.68 (0.39–1.18)		0.48 (0.27–0.84)	0.0104
Centre-Nord	396/40	10	0.68 (0.34–1.33)		0.61 (0.31–1.19)	0.144
Centre-Ouest	435/31	7.1	0.46 (0.25–0.86)		0.39 (0.21–0.72)	0.00275
Centre-Sud	177/17	9.4	0.63 (0.29–1.40)		0.55 (0.24–1.25)	0.152
Est	299/10	3.3	0.21 (0.08–0.53)		0.27 (0.11–0.65)	0.00356
Hauts-Bassins	371/81	21.8	1.70 (1.00–2.90)		0.84 (0.49–1.44)	0.532
Nord	263/42	16.1	1.17 (0.65–2.11)		0.87 (0.47–1.6)	0.652
Plateau-Central	247/29	11.6	0.80 (0.40–1.59)		0.69 (0.34–1.39)	0.298
Sahel	93/23	24.3	1.95 (1.16–3.28)		1.94 (1.09–3.45)	0.0246
Sud-Ouest	157/11	7.1	0.47 (0.21–1.02)		0.4 (0.18–0.88)	0.0236
Place of residence				*p* < 0.0001		
urban	1024/276	27	0.37 (0.31–0.44)		Ref	
rural	3000/300	10	0.30 (0.24–0.38)		0.6 (0.45–0.79)	< 0.001
Availability of electricity				*p* < 0.0001		
No	3333/400	12	0.14 (0.12–0.16)		Ref	
Yes	691/176	25.4	2.50 (1.93–3.23)		0.77 (0.53–1.13)	0.179
Age of the head of household				0.004		
< 25	125/8	6.2	0.06 (0.03–0.14)		Ref	
25–34	953/120	12.5	2.17 (0.99–4.78)		1.11 (0.52–2.4)	0.784
35–44	1287/227	17.7	3.25 (1.47–7.16)		1.25 (0.57–2.76)	0.576
45–54	801/112	14	2.46 (1.10–5.51)		1.1 (0.5–2.46)	0.81
55–64	422/61	14.4	2.54 (1.17–5.53)		1.18 (0.54–2.55)	0.683
65+	436/49	11.2	1.90 (0.81–4.46)		1.01 (0.44–2.32)	0.989
Food insecurity level				0.012		
Absent	1319/215	16.3	0.20 (0.16–0.23)		Ref	
Midle	944/153	16.3	0.99 (0.77–1.29)		1.27 (0.97–1.67)	0.0816
moderate	861/109	12.7	0.74 (0.54–1.01)		1.13 (0.82–1.57)	0.45
severe	875/95	10.8	0.62 (0.45–0.87)		1.16 (0.83–1.63)	0.386
Household size (members)				0.299		
1–2	21/5*	24.7	0.33 (0.10–1.08)			
3–4	626/94	14.9	0.54 (0.16–1.84)			
5–6	960/151	15.8	0.57 (0.17–1.92)			
≥ 7	2417/326	13.5	0.48 (0.14–1.58)			
Wealth Index				*p* < 0.0001		
Poorest	823/61	7.4	0.08 (0.06–0.11)		Ref	
Poorer	804/54	6.7	0.89 (0.55–1.44)		0.85 (0.52–1.41)	0.532
Middle	892/108	12.1	1.70 (1.12–2.59)		1.49 (0.95–2.34)	0.0787
Richer	779/141	18.1	2.75 (1.84–4.11)		2.12 (1.32–3.41)	0.00196
Richest	725/212	29.3	5.15 (3.47–7.64)		3.1 (1.74–5.53)	< 0.001
Sex of the head of household				0.384		
M	3589/507	14.1	0.16 (0.15–0.19)			
F	434/69	15.9	1.15 (0.84–1.58)			
women	*N*/*n*	Proportion (weighted)	OR95%CI	*p*-value Wald	Model II_aOR (95% CI)	*p* value
Currently working				0.0325		
No	1420/176	12.4	0.14 (0.12–0.17)			
Yes	2603/400	15.4	1.29 (1.02–1.62)			
Education level				4.85e- 6		
No formal education	2865/342	11.9	0.14 (0.12–0.16)		Ref	
primary	475/91	19.1	1.75 (1.29–2.37)		1.27 (0.9–1.8)	0.17
secondary	624/133	21.3	1.99 (1.51–2.63)		1.23 (0.88–1.72)	0.215
tertiary	60/10	17.2	1.53 (0.60–3.92)		0.52 (0.2–1.39)	0.192
Mother’s marital status				0.0093		
Never married	89/18	20.3	0.25 (0.13–0.51)		Ref	
married	2775/428	15.4	0.72 (0.35–1.47)		0.84 (0.38–1.86)	0.664
Living with a partner	1068/114	10.7	0.47 (0.24–0.94)		0.71 (0.32–1.55)	0.384
widowed	43/5	11.5	0.51 (0.14–1.83)		0.65 (0.16–2.57)	0.535
Separated	9/3*	31.2	1.79 (0.18–17.69)		0.7 (0.05–9.11)	0.788
No longer living with a partner	39/8	20.8	1.03 (0.35–3.08)		0.76 (0.24–2.4)	0.643
Age group				7.43e- 8		
15–19	194/6	3.1	0.13 (0.11–0.16)		Ref	
20–24	794/78	9.8	3.10 (1.76–5.44)		3,09 (1.22–7.85)	0.0178
25–29	1003/135	13.5	0.33 (0.17–0.62)		4.85 (1.94–12.08)	0.00652
30–34	878/161	18.3	1.50 (0.97–2.31)		6.45 (2.56–16.27)	0.0763
35–39	700/138	19.7	1.12 (0.76–1.66)		6.48 (2.53–16.61)	0.0001
40–44	355/43	12.2	1.38 (1.03–1.86)		4.64 (1.71–12.6)	0.0027
45–49	100/15	14.6	1.03 (0.84–1.25)		5.56 (2.02–15.32)	0.0010
Occupation type				7.37E-13		
notworking	1292/159	12.3	0.14 (0.11–0.17)		Ref	
professional manager	107/41	38.2	4.40 (2.58–7.52)		1.95 (1.07–3.56)	0.0293
clerical	19/5*	27.9	2.75 (0.64–11.82)		1.16 (0.32–4.23)	0.823
Sales	757/146	19.3	1.69 (1.28–2.24)		1.24 (0.92–1.67)	0.149
agricultural employee	1539/157	10.2	0.81 (0.61–1.08)		1.29 (0.94–1.77)	0.109
domestic employee	57/15	25.4	2.43 (1.02–5.77)		1.44 (0.66–3.11)	0.359
service	53/6	10.6	0.84 (0.33–2.14)		0.48 (0.19–1.26)	0.137
skilled manual	140/31	22.5	2.06 (1.23–3.44)		1.35 (0.81–2.26)	0.246
unskilled	51/16	31.2	3.22 (1.50–6.95)		2.53 (1.23–5.22)	0.0122
Number of living children				0.077		
0	0	NA				
1	689/67	9.7	0.11 (0.08–0.15)			
2	757/113	14.9	1.64 (1.06–2.52)			
3 to 4	1500/224	14.9	1.64 (1.09–2.46)			
5 to 6	815/134	16.5	1.84 (1.22–2.79)			
≥ 7	262/39	14.8	1.62 (0.97–2.72)			
Dietary diversity score (DDS)				0.246		
< 5 food groups	129/22	16.7	0.20 (0.12–0.32)			
>= 5 food groups	211/48	22.9	1.48 (0.76–2.89)			
Children				0.039		
Age group						
6–12	557/83	14.9	0.18 (0.14–0.23)			
12–24	1073/156	14.6	0.97 (0.71–1.32)			
24–36	795/138	17.3	1.19 (0.86–1.66)			
36–59	1599/199	12.4	0.81 (0.62–1.05)	0.504		
Child Sex						
Male	2067/304	14.7	0.17 (0.15–0.20)			
Female	1957/272	13.9	0.94 (0.77–1.13)	0.00539		
Birth’s Rank						
1	842/78	9.2	0.10 (0.07–0.14)			
2	701/105	15	1.74 (1.19–2.55)			
3	692/105	15.2	1.76 (1.19–2.60)			
≥ 4	1788/288	16.1	1.89 (1.33–2.68)	0.235		
Diarrhea in the last two weeks						
No	3322/466	14	0.16 (0.14–0.18)			
Yes	697/111	15.9	1.16 (0.91–1.47)	0.138		
Fever in the last two weeks						
No	2976/409	13.8	0.16 (0.14–0.18)			
Yes	1044/167	16	1.19 (0.95–1.50)	0.447		
Vitamin A during the last 6 months						
No	1204/192	16	0.19 (0.16–0.23)			
Yes	1084/159	14.7	0.91 (0.70–1.17)			
Breastfeeding				0.0062		
Ever breastfed, not currently breastfeeding	577/111	19.2	0.24 (0.19–0.30)			
Still breastfeeding	268/42	15.8	0.79 (0.52–1.20)			
Never breastfed	1476/199	13.5	0.65 (0.50–0.85)			

*DBM* Double burden of malnutrition. N/n denotes the number of mother-child pair with the specified condition (n) divided by the total number of pairs in the corresponding category (N). Percentages are weighted to account for the survey design. *OR *Odds ratio, *95% CI*  95% confidence interval, *OR* Odds ratio, *AoR* Adjusted odds ratio, *95% CI C*onfidence interval. The *p*-value (Wald) represents the significance of the Wald chi-square test. Model I adjusts for women’s sociodemographic characteristics. Model II builds on Model I by incorporating household-level characteristics. Model II further includes child-related variables. Model IV adds women’s dietary diversity to the preceding covariates. The *P *value refers to the significance of each covariate within its model; *p* < 0.05 was considered statistically significant

^1^Model II: best fit model

At the household level, significant associations were found with region (*p* < 0.0001), type of residence (*p* < 0.0001), electricity availability (*p* < 0.001), age of the household head (*p* = 0.004), household food security (*p* = 0.018), and wealth index (*p* < 0.001).

Among the maternal characteristics, current employment status (*p* = 0.027), educational level (*p* = 0.015), marital status (*p* = 0.0210), age group (*p* = 0.034), number of living children (*p* = 0.015), and type of occupation (*p* = 0.048) were significantly associated.

For child-related characteristics, significant associations were found with age group (*p* = 0.002), birth rank (*p* = 0.039) and breastfeeding status (*p* = 0.017).

Model II was the best fit for the data. Adding household-level variables to Model I significantly improved the model fit (LRT = 136.32, *p* < 0.001). Model III, which incorporates child-specific characteristics (age, breastfeeding, recent fever), did not significantly improve the fit compared with Model II (*p* = 0.68), suggesting limited explanatory value. Model IV, adding maternal dietary diversity, also failed to improve the model (*p* = 0.26).

After adjustment (Model II), several factors remained independently associated with DBM.

At the household level, those in Centre Est (aOR = 0.44, 95% CI: 0.23–0.85, *p* = 0.015) and Est (aOR = 0.30, 95% CI: 0.11–0.77, *p* = 0.013) had significantly lower odds, whereas those in Sahel had higher odds (aOR = 2.83, 95% CI: 1.53–5.44, *p* = 0.001). Place of residence remained a strong independent risk factor: the prevalence of DBM was 27% in urban households versus 10% in rural households, and rural households were significantly less likely to experience DBM than urban households were (aOR = 0.53, 95% CI: 0.37–0.76, *p* < 0.001). A clear socioeconomic gradient persisted, with the wealth index showing increased odds across quintiles: compared with the poorest households, middle (aOR = 1.67, 95% CI: 1.02–2.74, *p* = 0.041) and richer households (aOR = 1.93, 95% CI: 1.13–3.31, *p* = 0.01) had significantly greater odds of DBM.

Maternal age was one of the strongest predictors of DBM. The prevalence rose steadily with age, from 3.1% in adolescents (15–19 years) to nearly 20% in women aged 30–39 years. After adjustment, compared with those of the youngest group (15–19 years), the odds of DBM were three times greater among women aged 20–24 years (OR = 3.09, 95% CI = 1.22–7.85), nearly five times greater in the 25–29 years (aOR = 4.85, 95% CI = 1.94–12.08), and six times greater in the 30–39 years (aOR = 6.45–6.48). Elevated odds also persisted in women aged 40–44 years (aOR = 4.64, 95%: 1,71–12,6) and 45–49 years (aOR = 5.56, 95% CI: 2.02–15.32).

After adjustment, women in professional/managerial occupations, where the DBM prevalence reached 38.2%, remained significantly more likely to experience DBM (aOR = 1.95, 95% CI: 1.07–3.56, *p* = 0.029). Similarly, women in unskilled occupations, with a prevalence of 31.2%, also presented significantly higher odds (aOR = 2.53, 95% CI: 1.23–5.22, *p* = 0.012) than non-working women did (12.3%).

### Sensitivity analysis

The sensitivity analysis confirmed the robustness of our findings. Of 56 estimated coefficients, 54 (96.4%) maintained consistent direction and statistical significance. DBM 4 prevalence remained stable (15.5% vs. 14.9%). Two associations (maternal age 35–39 years, Sud Ouest regions) lost statistical significance in the restricted sample (Annex 3).

## Discussion

This study aimed to estimate the prevalence of different forms of double burden of malnutrition (DBM) and to identify factors associated with the most frequent combination. The mother child approach highlights the coexistence of contrasting outcomes within the same household, reflecting inequalities in resources allocation, feeding practices and exposure to health risk. This issue is particularly critical in LMIC undergoing rapid nutrition transition, challenging traditional assumptions that link malnutrition solely to poverty [6]. In this context, examining these conditions separately limits our understanding of nutritional dynamics, whereas a joint analysis help uncover complex and often hidden inequalities [4]. As such, household level analysis of DBM provides an essential and complementary perspective to individual and population level approaches.

By identifying the most prevalent DBM profile and characteristics associated, this study makes an important contribution to the global health nutrition by providing an integrated perspective that captures both individual factors and shared household environments. It also generates actionable evidence to inform health and nutrition policies.

Our study population demonstrate an approximate one third urban residence, which closely aligns Burkina Faso national demographic profile. According to 2019 population and d housing census, around 26% of the population resides in urban area [24], confirming that our sample composition accurately reflects the country’s true distribution.

Using nationally representative the country DHS 2021, we assessed the prevalence of different combinations of malnutrition at both the individual and household levels (mother-child), as well the characteristics associated with of the most prevalent combination.

The most frequent DBM combination was the coexistence of child anemia and maternal overweight/obesity within a single household, which affects 14.3% of households, followed by anemia and overweight/obesity in women at the individual level (9.5%) and child stunting with maternal overweight/obesity at the household level (3.6%). After adjustment, independent factors associated with the most prevalent DBM included urban residence, household wealth, maternal age, and maternal occupation, with strong regional disparities observed.

### High burden of anemia among children and rising burden of overweight and obesity in women

In our study, anemia emerged as the most prevalent nutritional disorder, affecting 72% of children and 53.9% of women of reproductive age, indicating a severe public health problem according to WHO thresholds.

Among children, this prevalence exceeds the sub-Saharan average (64%) reported across 34 countries [32] and is comparable to the high burden observed in South Asian, particularly in India (67%) [33]. Globally, anemia remains disproportionally concentrated in low and middle income countries, where prevalence reaches 67.4%, compared with 14.4% in high income settings [34].

Among women in reproductive age, the prevalence of anemia aligns with regional estimates for sub Saharan Africa( 48–60%) [35]. While comparable to levels reported in South Asia [33], it remains substantially higher than those observed in high income regions (< 22%) [36].

These findings underscore the persistence of anemia as a structural public health challenge in Burkina Faso. The high burden likely reflects the combined effects of malaria endemicity, food insecurity, inadequate dietary intake, and reproductive factors such as high parity, compounded by limited access to quality maternal health service [37, 38].

Our findings revealed that 25.4% of women were living with overweight or obesity, representing a 128% relative increase compared with Burkina Faso DHS 2010 [39]. In a recent multi-country study including nine sub-Saharan countries, reported that 34.79% of women were effected by overnutrition, with 21.81% overweight and 12.99% obese [40]. Previous analyses have shown that the prevalence of overnutrition in women nearly tripled between 1991 and 2014 [41]. Our findings are consistent with broader trends documented in DHS-based analyses across sub-Saharan Africa, although variations exist between countries [12, 28, 41, 42]. In many SSA contexts, urban women and those from wealthier households appear to be the most affected groups [12, 43].

This growing burden of overweight/obesity is emblematic of the nutrition transition, which is marked by dietary shifts toward energy-dense foods, reduced physical activity, and increased urbanization. Projections suggest that the prevalence of overweight and obesity will double by 2050, with sub‑Saharan Africa contributing the largest share to this increase [2].

### Multiple types of the double burden of malnutrition (DBM)

Our findings indicate an alarming prevalence of the double burden of malnutrition (DBM) in Burkina. When all the forms were combined across the household and individual levels, the overall weighted prevalence reached 30%, indicating that nearly one in three households or individuals experienced the coexistence of undernutrition and overnutrition. At the household level, 20% of households were affected by at least one form of DBM, underscoring the growing complexity of the nutrition landscape.

The most frequent combination was children with anemia, with the prevalence of maternal overweight reaching 14.32%, which has nearly doubled since 2010 (7.4%) [14], illustrating the accelerating pace of nutrition-related changes in the country. The coexistence of child anemia and maternal overnutrition observed in this study reflects a pattern described across Africa [10, 11, 14].

Although few national studies have quantified DBM precisely, prior urban-based research among adults in Burkina Faso reported a prevalence of 23.5% associated with factors such as high income, female sex and physical inactivity, although the scale of the study was restricted to urban adults [17]. Similarly, a study in Ouagadougou among schoolchildren reported stunting (8.1%),wasting (8.7%) and overweight/obesity (4.4%),reflecting the coexistence of contrasting nutritional problems within the same population [18].

Our estimates are consistent with 49 multicounty analyses across sub-Saharan Africa, indicating a pooled prevalence of DBM of 16.2%, ranging from 3.1% in Ethiopia to 42.2% in South Africa [14], and contrast with higher levels observed in North Africa, up to 24% [13], where the nutrition transition is more advanced. Lower prevalence rates were observed in other context [10, 44].

The child with overweight and mother with anemia profile remained rare (0.65%), suggesting distinct underlying dynamics: child overweight is still uncommon where undernutrition persists, and maternal anemia tends to coincide with child stunting or wasting rather than overweight [14].

### Characteristics associated with the most common form of DBM (children with anemia and women with overweight/obesity)

Our findings demonstrate that the coexistence of child with anemia and mother with overweight/obesity is shaped by a complex interplay of household, and individua level characteristics. Significant independent’s associated risk factors included region, place of residence, household wealth index level, maternal age and women’s employment.

Urban residence was identified as a significant risk factor for the DBM, with rural households being 40% less likely to experience DBM than urban households. This finding supports the concept of urban “obesogenic environments”, characterised by increased availability and consumption of ultra processed, energy dense foods, reduced physical activity, and time constraints that negatively influence childcare and feeding practices [10, 20]. These conditions promote overweight and obesity among women while failing to improve diet quality, creating a nutritional mismatch within households. At the same time, rural settings may retain more traditional food systems and higher physical activity, offering partial protect against this DBM, as also suggested in a previous study [17] .

This imbalance is reflected in the high burden of anemia among both children and mothers, likely driven by poor diet quality, limited dietary diversity, and recurrent infections [44]. Notably, the majority of women (65%) in our study had inadequate dietary diversity, highlighting that increased food access does not necessarily translate into improved micronutrient intake. Instead, caloric sufficiency, particularly in urban and transitioning contexts [11].

A clear socioeconomic gradient was observed, with the odds of DBM increasing across wealth quintiles: households in the middle quintile were approximately 67% more likely to experience DBM, while richer households had 93% higher odds compared with the poorest. This paradox, well documented [11, 13, 14], and reflects an early or intermediate stage of nutrition transition [6]. At this stage, higher income tends to increase access to energy dense but nutrient poor foods and more sedentary lifestyles, especially among women, without ensuring improvements in child nutrition. As shown in [14], although greater household wealth is strongly maternal associated with maternal overweight, childhood anemia often continues regardless of economic conditions, largely driven by environmental factors, infections, and inadequate feeding practices.

Regional disparities were pronounced. Households in Centre Est were 56% less likely and those in Est 70% less likely to experience DBM, whereas those in the Sahel region had 183% higher odds. Variations across regions likely reflect differences in urbanisations, food environments, cultural practice and access to health services. Evidence suggests that the association between urbanisation and DBM is particularly strong in countries at earlier stages of the nutrition transition [14]. Regions with lower DBM risk may remain at earlier stages, while more urbanised or economically dynamic regions are more exposed to rapid dietary and lifestyle changes, reinforcing the coexistence of overnutrition and undernutrition.

Maternal related factors also play a key role. The risk of DBM increases with maternal age, likely reflecting cumulative exposure to obesogenic environments and age-related metabolic changes that favour overweight and obesity. Women’s employment may also have a dual effect: while it can improve household income, it may also reduce time for childcare and optimal feeding practices, potentially increasing reliance on processed foods and limiting engagement with preventive care [14].

Overall, DBM in the context appears to result from the interaction of three main mechanisms: (i) dietary shift toward energy dense but micronutrient poor food [20]; ii) persistent exposure to infections that exacerbate micronutrient deficiencies [45] and iii) socio-behavioural constraints affecting caregiving and food allocation.

In contrast to more advanced stages of the nutrition transition observed in Latin American countries [22, 46, 47], where DBM is concentrated among poorer populations, our findings suggest that DBM remains more prevalent among poorer populations, our findings suggest that DBM remains more prevalent among wealthier and urban households. This pattern reflects an ongoing and incomplete transition in Sub-Saharan Africa [14, 17], where urbanisation and increasing wealth act as catalysts for maternal overweight/obesity,, while structural and environmental constraints sustain anemia.

## Conclusion

Our analysis of national representative data from the 2021 Burkina Faso Demographic and Health Survey reveals that the double burden of malnutrition is not a theoretical concern but rather a lived reality for one in seven households. The most prevalent form, which is a child with anemia co-occurring alongside mother with overweight or obesity, highlights the paradoxical nutritional transition, wherein nutritional deficiencies and excesses coexist within the same family unit. These patterns were especially pronounced among households in urban settings, those with higher socioeconomic status, and mothers who were older or engaged in professional occupations. These associations underscore the intersection of demographic, economic and occupational factors with dietary practices and health behaviours.

In addition to statistics, the human cost of this dual burden is profound. Children with anemia face risk to cognitive development and immune function, whereas overweight mothers are at greater risk of chronic disease, creating an intergenerational cycle of poor health. Addressing DBM, therefore, requires integrated interventions that simultaneously address undernutrition and overnutrition. Policies must promote access to rich foods, support mothers a.

nd caregivers through targeted education and regulate environments that foster poor dietary habits. In addition, strategies should be tailored to regional and socioeconomic contexts, acknowledging the disparities revealed in our analysis.

By providing evidence of the magnitude and associated characteristics with DBMs in Burkina Faso, this study points to urgent priorities for public health. Preventing and reversing the double burden of malnutrition is not only central to advancing child and maternal health but also indispensable for achieving sustainable development goals related to nutrition, health and equity.

### Interpretation and implications

The findings that wealthier and urban households are at greater risk of DBM highlight an ongoing nutrition transition in Burkina Faso and sub-Saharan Africa [14]. The 14.3% prevalence of child anemia with maternal overweight, within an overall 30% DBM prevalence, indicates an intermediate, inequitable stage of this transition, where maternal overnutrition coexists with persistent child undernutrition.

Programmatically, these results call for “double-duty” actions [4] that simultaneously target maternal overweight and child undernutrition while addressing broader structural drivers such as poverty, urbanization, and food environments. Interventions promoting balanced diets, infection control, iron-rich foods, and time-efficient healthy meal preparation for working women are essential.

Future research should adopt mixed methods approaches to better understand how the DBM is shaped within household. In particular, qualitative studies could help unpack intra household dynamics, including food allocation and caregiving practices. Such approaches would ensure that future evidence remains context specific and grounded in lived experiences.

Importantly, our findings suggest that DBM is not confined to the most disadvantaged groups. Future research should therefore place greater emphasis on urban settings, examining why and how nutrition transition affects population subgroups differently affects within urban settings.

Longitudinal research would further help to clarify how these patterns develop over time, particularly the role of cumulative exposure to cumulative exposure to changing environments in shaping women’s and children’s nutritional status. Finally, future research should maintain a strong equity-oriented perspective, while acknowledging that vulnerabilities are evolving and may increasingly affect non-traditional risk group.

### Limitations and strengths

Several limitations should be acknowledged. First, although DHS data are nationally representative and households are randomly selected, anthropometric measurements are collected on a subsample of households. As a result, some missing values likely reflect structural non eligibility rather than true missingness. In addition, restricting the analysis to children with valid measurements may have introduced selection bias and reduced generalisability.

Second, the hierarchical structure of the data may lead to non-independence of observations. While survey weighted regression models account for clustering and sampling design, some residual intra household correlation may persist.

Third, the cross-sectional design precludes any causal inference. Furthermore, some DBM combinations were relatively rare, limiting statistical power of subgroup analyses. The DHS also does not allow for a detailed investigation of the underlying cause of anemia.

Finally, the selection of specific DBM outcome (child with anemia and mother with overweight/obesity), although justified by its prevalence and public health relevance, was data driven and may limit comparability with other studies.

Despite these limitations, this study benefits from nationally representative data, rigorous handling of the complex survey design, and a comprehensive assessment of multiple forms of the DBM, providing timely and relevant evidence in Burkina Faso context.

A potential limitation was the inclusion of mothers with multiple children (22.7%), which could introduce within mother correlation. However, our sensitivity maintained identical patterns, and DBM4 prevalence remained stable. The two associations losing significance likely reflected statistical power rather than bias. These findings support the validity of our analytical approach.

## Supplementary Information

Supplementary Material 1.

Supplementary Material 2.

## Data Availability

The authors used data from the Demographic and Health Surveys, which are publicly available and can be accessed from https://dhsprogram.com.

## Abbreviations

aOR: Adjusted Odds Ratio
CI: Confidence Interval
DBM: Double Burden of Malnutrition
DHS: Demographic and Health Survey
OR: Odd Ratio
WHO: World Health Organization
UNICEF: United Nation Children’s Fund
