# Supplementary material for: When undernutrition meets overweight: prevalence and determinants of double burden of malnutrition among women-child pairs in Burkina Faso from the 2021 Demographic and Health Survey
Source: BMC Public Health. 2026 May 2;26:2789. doi: 10.1186/s12889-026-27603-x (PMC13637117; doi:10.1186/s12889-026-27603-x)
Supplement: Supplementary file 1 [file 12889_2026_27603_MOESM1_ESM.docx]

**Supplement materials**

**Assessment of food insecurity scale (FIES)**

Food insecurity was assessed via an adapted Food Insecurity Experience Scale (FIES), which is based on eight specific questions asked at the household level over the previous 12 months.

For FEIS, the following questions were asked: 1) Worrying about not having enough food due to a lack of money or resources? 2) Unable to eat healthy and nutritious food because of a lack of resources? 3) Ate only a few kinds of foods because of a lack of resources? 4) Had one skipped a meal because there was not enough money or resources? 5) Ate less than needed because of a lack of resources? 6) Run out of food due to lack of money or resources? 7) Felt hungry but did not eat because of a lack of resources? and 8) went a whole day and night without eating because of a lack of money or other resources. Each affirmative ("Yes") response was coded as 1, and each negative ("No") response was coded as 0. A composite score ranging from 0 to 8 was calculated. Households were then categorized based on their total score: 0 points (food insecurity absent), 1-3 points (mild food insecurity), 4 points (moderate food insecurity) and 4–8 points for severe food insecurity(15).

**Assessment of minimum dietary diversity (MDD)**

Minimum dietary diversity (MDD) for women, which is a standardized measure developed by the Food and Agriculture Organization (FAO), was included to assess the micronutrient adequacy of diets among women of reproductive age (15–49 years). It is based on the consumption of ten specific food groups within the previous 24 hours. The different food groups are as follows: 1-Food-based cereals; 2-Roots white/pale, pale containing starch, tubercles and plantains; 3-Beans, peas lentils; 4-Nuts and seeds; 5- Milk, cheese yogurt other products dairy products; 6-meat, fish, chicken, poultry offal; 7-Eggs vegetables leafy green leaves; 8-Other fruits and vegetables rich in vitamin A; 9-Other vegetables; and 10- Other fruits.

Each food group consumed is assigned a score of 1; those not consumed receive a score of 0. The scores are summed up to obtain a total dietary diversity score ranging from 0 to 10. The MDD indicator classifies women as having achieved minimum dietary diversity if they have consumed at least five out of the ten food groups in the past 24 hours. Minimum dietary diversity (MDD): consumed at least 5 food groups out of 10 (yes/no) (15).
