# Supplementary material for: When undernutrition meets overweight: prevalence and determinants of double burden of malnutrition among women-child pairs in Burkina Faso from the 2021 Demographic and Health Survey
Source: BMC Public Health. 2026 May 2;26:2789. doi: 10.1186/s12889-026-27603-x (PMC13637117; doi:10.1186/s12889-026-27603-x)
Supplement: Supplementary file 2 [file 12889_2026_27603_MOESM2_ESM.docx]

**Annex**

**Section Result**

**Model comparisons**

Annex 1 reports the results of sequential model comparisons via the Rao–Scott likelihood ratio test, where each model was assessed against its immediate predecessor. The associated 2logLR values and p values indicate whether the inclusion of additional predictors significantly improved model fit. In addition, pseudo-R² measures (McFadden, Cox & Snell, and Nagelkerke), computed from model deviances, are presented to provide an estimate of the proportion of variance explained. As shown in Table 7, the comparison between Model I and Model II indicated a highly significant improvement in model fit (2logLR=136.3, p< 0.0001). In contrast, the inclusion of additional predictors in Models III and IV did not yield statistically significant improvements (p=0.68 and p=0.26, respectively). In terms of explanatory power, the Nagelkerke pseudo R2 increased from 0.09 in Model I to 0.16 in Model II and 0.22 in Model III, reaching 0.68 in Model IV. Although Model IV displayed the highest explanatory capacity, Model II emerged as the most parsimonious and statistically robust specification.

**Annex 1: Model comparison based on likelihood ratio tests and pseudo-R² indices**

| **Model Comparison** | **-2 Log-Likelihood (2logLR)** | **Wald P value** | **Mc-Fadden R²** | **Cox & Snell R²** | **Nagelkerke R²** |
| --- | --- | --- | --- | --- | --- |
| Model I | Ref | Ref | 0.062 | 0.05 | 0.089 |
| Model II | 136.32 | < 0.0001 | 0.117 | 0.092 | 0.164 |
| Model III | 3.1 | 0.682 | 0.162 | 0.128 | 0.224 |
| Model IV | 1.29 | 0.263 | 0.561 | 0.423 | 0.677 |

**Annex 2: Stepwise Multivariate analysis with the other 3 models**

|  | **Model I_OR (95% CI)** | **p value** | **Model III_OR (95% CI)** | **p value** | **Model IV_OR (95% CI)** | **p value** |
| --- | --- | --- | --- | --- | --- | --- |
| **Household** |  |  |  |  |  |  |
| **Region** |  |  |  |  |  |  |
| Boucle du Mouhoun |  |  | Ref |  | Ref |  |
| Cascades |  |  | 1.05 (0.44–2.5) | 0.92 | 1.73 (0.05–66.45) | 0.765 |
| Centre |  |  | 1.13 (0.6–2.14) | 0.696 | 22.73 (0.42–1233.74) | 0.123 |
| Centre-Est |  |  | 0.44 (0.23–0.85) | 0.0154 | 0 (0–0) | <0.001 |
| Centre-Nord |  |  | 0.67 (0.31–1.48) | 0.322 | 0 (0–0) | <0.001 |
| Centre-Ouest |  |  | 0.58 (0.28–1.21) | 0.147 | 1.1 (0.02–76.75) | 0.963 |
| Centre-Sud |  |  | 0.72 (0.28–1.85) | 0.496 | 0.02 (0–2.3) | 0.106 |
| Est |  |  | 0.3 (0.11–0.77) | 0.0131 | 0.22 (0–25.88) | 0.528 |
| Hauts-Bassins |  |  | 0.87 (0.44–1.7) | 0.677 | 0.49 (0.02–13.9) | 0.674 |
| Nord |  |  | 0.91 (0.44–1.87) | 0.796 | 23.01 (0.24–2182.52) | 0.173 |
| Plateau-Central |  |  | 0.65 (0.3–1.42) | 0.283 | 3.94 (0.04–390.02) | 0.553 |
| Sahel |  |  | 2.89 (1.53–5.44) | 0.00107 | 0.19 (0.01–3.55) | 0.258 |
| Sud-Ouest |  |  | 0.4 (0.16–1) | 0.0488 | 0 (0–0) | <0.001 |
| **Place of residence** |  |  |  |  |  |  |
| urban |  |  |  |  |  |  |
| rural |  |  | 0.53 (0.37–0.76) | <0.001 | 0.03 (0–0.47) | 0.0143 |
| **Availability of electricity** |  |  |  |  |  |  |
| No |  |  | Ref |  | Ref |  |
| Yes |  |  | 0.83 (0.55–1.27) | 0.394 | 5.48 (0.36–83.36) | 0.216 |
| **Age of the head of household** |  |  |  |  |  |  |
| < 25 |  |  | Ref |  | Ref |  |
| 25-34 |  |  | 1.43 (0.43–4.72) | 0.558 | 0.01 (0–0.42) | 0.0172 |
| 35-44 |  |  | 1.83 (0.55–6.12) | 0.323 | 0.01 (0–0.34) | 0.0135 |
| 45-54 |  |  | 1.54 (0.45–5.22) | 0.49 | 0.01 (0–0.37) | 0.015 |
| 55-64 |  |  | 1.32 (0.37–4.67) | 0.67 | 0.01 (0–0.72) | 0.0351 |
| 65+ |  |  | 1.18 (0.34–4.09) | 0.799 | 0 (0–0.59) | 0.0345 |
| **Food insecurity level** |  |  |  |  |  |  |
| Absente |  |  |  |  |  |  |
| Midle |  |  | 1.34 (0.92–1.94) | 0.124 | 2.18 (0.34–14.05) | 0.407 |
| moderate |  |  | 1.36 (0.92–2.02) | 0.126 | 6.31 (0.62–64.01) | 0.117 |
| severe |  |  | 0.98 (0.63–1.53) | 0.925 | 4.57 (0.48–43.23) | 0.181 |
| **Household size (members)** |  |  |  |  |  |  |
| 1-2 |  |  |  |  |  |  |
| 3-4 |  |  |  |  |  |  |
| 5-6 |  |  |  |  |  |  |
| ≥ 7 |  |  |  |  |  |  |
| **Wealth Index** |  |  |  |  |  |  |
| Poorest |  |  | Ref |  | Ref |  |
| Poorer |  |  | 0.87 (0.5–1.5) | 0.611 | 0.05 (0–0.92) | 0.0442 |
| Middle |  |  | 1.67 (1.02–2.74) | 0.0413 | 0.26 (0.03–2.14) | 0.206 |
| Richer |  |  | 1.93 (1.13–3.31) | 0.0171 | 2 (0.07–60.26) | 0.685 |
| Richest |  |  | 2.93 (1.49–5.74) | 0.00186 | 0.08 (0–3.18) | 0.175 |
| **Sex of the head of household** |  |  |  |  |  |  |
| M |  |  |  |  |  |  |
| F |  |  |  |  |  |  |
| **women** |  |  |  |  |  |  |
| **Currently working** |  |  |  |  |  |  |
| No |  |  |  |  |  |  |
| Yes |  |  |  |  |  |  |
| **Education level** |  |  |  |  |  |  |
| no formal education | Ref |  |  |  |  |  |
| primary | 1.73 (1.25–2.41) | 0.00111 | 1.27 (0.85–1.91) | 0.244 | 0.1 (0.01–1.19) | 0.0677 |
| secondary | 1.86 (1.34–2.59) | <0.001 | 1.38 (0.94–2.02) | 0.101 | 0.1 (0.01–0.64) | 0.0166 |
| tertiary | 0.89 (0.33–2.34) | 0.806 | 0.32 (0.11–0.92) | 0.0351 | 0.07 (0–1309.2) | 0.595 |
| **Mother's marital status** |  |  |  |  |  |  |
| Never married | Ref |  | Ref |  |  |  |
| maried | 0.77 (0.36–1.66) | 0.511 | 0.67 (0.25–1.77) | 0.414 |  | <0.001 |
| livingwithpart | 0.53 (0.25–1.12) | 0.0954 | 0.49 (0.18–1.33) | 0.16 |  | <0.001 |
| widwoded | 0.49 (0.13–1.87) | 0.293 | 0.28 (0.04–1.82) | 0.181 |  | <0.001 |
| Separated | 0.95 (0.07–12.13) | 0.966 | 0.12 (0–5.56) | 0.278 | 3.71 (0.01–1728.26) | 0.671 |
| No longer living with a partner | 0.73 (0.24–2.27) | 0.59 | 0.27 (0.06–1.26) | 0.0942 |  |  |
| **Age group** |  |  |  |  |  |  |
| 15-19 |  |  |  |  |  |  |
| 20-24 | 3.89 (2.13–7.08) | <0.001 | 6.15 (2.68–14.1) | <0.001 |  | <0.001 |
| 25-29 | 0.36 (0.18–0.71) | 0.00309 | 0.49 (0.23–1.04) | 0.0632 | 2.62 (0.27–25.62) | 0.402 |
| 30-34 | 1.6 (1.02–2.52) | 0.0409 | 2.35 (1.3–4.26) | 0.00499 |  | <0.001 |
| 35-39 | 1.17 (0.78–1.76) | 0.444 | 1.32 (0.78–2.23) | 0.298 | 1.06 (0.15–7.33) | 0.951 |
| 40-44 | 1.26 (0.93–1.7) | 0.142 | 1.17 (0.78–1.75) | 0.446 |  | <0.001 |
| 45-49 | 0.99 (0.8–1.22) | 0.936 | 0.98 (0.73–1.3) | 0.866 | 0.2 (0.03–1.55) | 0.122 |
| **Occupation type** |  |  |  |  |  |  |
| notworking | Ref |  | Ref |  | Ref |  |
| professional manager | 2.71 (1.5–4.87) | <0.001 | 1.84 (0.78–4.33) | 0.161 | 2.66 (0.01–995.94) | 0.742 |
| clarical | 1.71 (0.44–6.69) | 0.44 | 0.78 (0.13–4.59) | 0.78 | 0 (0–0) | <0.001 |
| Sales | 1.44 (1.08–1.91) | 0.0137 | 1.59 (1.1–2.32) | 0.0149 | 0.36 (0.05–2.74) | 0.318 |
| agri employee | 0.82 (0.61–1.09) | 0.167 | 1.43 (0.98–2.09) | 0.0629 | 0.52 (0.03–10.35) | 0.664 |
| domestic employee | 2.18 (1.02–4.65) | 0.0431 | 1.52 (0.62–3.72) | 0.363 |  | <0.001 |
| service | 0.63 (0.26–1.55) | 0.316 | 0.49 (0.13–1.86) | 0.293 | 0 (0–0) | <0.001 |
| skilled manual | 1.82 (1.1–3) | 0.0193 | 1.06 (0.53–2.1) | 0.867 | 8.05 (0.59–109.11) | 0.115 |
| unskilled | 2.64 (1.27–5.49) | 0.00937 | 2.98 (1.04–8.5) | 0.0415 | 0.35 (0.02–6.86) | 0.483 |
| **number of living children** |  |  |  |  |  |  |
| 0 |  |  |  |  |  |  |
| 1 |  |  |  |  |  |  |
| 2 |  |  |  |  |  |  |
| 3 to 4 |  |  |  |  |  |  |
| 5 to 6 |  |  |  |  |  |  |
| ≥ 7 |  |  |  |  |  |  |
| **Dietary diversity score (DDS)** |  |  |  |  |  |  |
| < 5 food groups |  |  |  |  | Ref |  |
| >= 5 food groups |  |  |  |  | 2.77 (0.45–17) | 0.266 |
|  |  |  |  |  |  |  |
| **Children** |  |  |  |  |  |  |
| **Age group** |  |  |  |  |  |  |
| 6-12 |  |  |  |  |  |  |
| 12-24 |  |  | 0.98 (0.69–1.38) | 0.888 | 2.97 (0.08–106.39) | 0.545 |
| 24-36 |  |  | 0.76 (0.46–1.26) | 0.287 | 1.12 (0.01–140.76) | 0.963 |
| 36-59 |  |  |  |  |  |  |
| **Child Sex** |  |  |  |  |  |  |
| Male |  |  |  |  |  |  |
| Female |  |  |  |  |  |  |
| **Birth's Rank** |  |  |  |  |  |  |
| 1 |  |  |  |  |  |  |
| 2 |  |  |  |  |  |  |
| 3 |  |  |  |  |  |  |
| ≥ 4 |  |  |  |  |  |  |
| **Diarrhea in the last two weeks** |  |  |  |  |  |  |
| No |  |  |  |  |  |  |
| Yes |  |  |  |  |  |  |
| **Fever in the last two weeks** |  |  |  |  |  |  |
| No |  |  | Ref |  |  |  |
| Yes |  |  | 1.12 (0.82–1.54) | 0.474 | 2.76 (0.5–15.08) | 0.237 |
| **Vitamin A during the last 6 months** |  |  |  |  |  |  |
| No |  |  |  |  |  |  |
| Yes |  |  |  |  |  |  |
| **Breastfeeding** |  |  | Ref |  |  |  |
| Ever breastfed, not currently breastfeeding. |  |  |  |  |  |  |
| Still breastfeeding |  |  | 0.71 (0.47–1.09) | 0.122 | 1.08 (0.07–16.95) | 0.953 |
| Never breastfed |  |  | 0.85 (0.52–1.39) | 0.513 | 4.62 (0.62–34.58) | 0.133 |
|  |  |  |  |  |  |  |

**Annex 3: Sensitivity analysis comparing main and restricted samples (summary)**

|  | Main sample(n=4114) | Restricted sample†(n=3352)*^1^* |  |
| --- | --- | --- | --- |
| **Context** |  |  |  |
| Rural residence | 0.39 (0.28–0.54)*** | 0.40 (0.28–0.57)*** | ✓ |
| Region: Sahel vs Boucle du Mouhoun | 2.30 (1.24–4.27)** | 2.66 (1.42–4.99)** | ✓ |
| Region: Est vs Boucle du Mouhoun | 0.23 (0.09–0.60)** | 0.17 (0.06–0.51)** | ✓ |
| Region: Sud-Ouest vs Boucle du Mouhoun | 0.38 (0.16–0.90)* | 0.42 (0.18–1.02) | ✗ |
| **Household** |  |  |  |
| Has electricity | 1.24 (0.89–1.71) | 1.22 (0.85–1.75) | ✓ |
| Food insecurity: Severe vs Absent/Mild | 0.79 (0.51–1.22) | 0.79 (0.49–1.26) | ✓ |
| **Maternal** |  |  |  |
| Age 25-29 vs 15-19 years | 3.72 (1.24–11.19)* | 3.83 (1.17–12.56)* | ✓ |
| Age 30-34 vs 15-19 years | 4.79 (1.59–14.41)** | 4.26 (1.29–14.10)* | ✓ |
| Age 35-39 vs 15-19 years | 3.65 (1.13–11.77)* | 2.80 (0.78–10.13) | ✗ |
| Age 45-49 vs 15-19 years | 7.05 (1.80–27.63)** | 5.53 (1.26–24.30)* | ✓ |
| Secondary vs No education | 1.74 (1.15–2.63)** | 1.75 (1.14–2.69)* | ✓ |
| Parity 2 vs 1 | 2.05 (1.15–3.64)* | 2.10 (1.20–3.69)** | ✓ |
| Parity 5-6 vs 1 | 2.80 (1.37–5.71)** | 3.53 (1.69–7.35)*** | ✓ |
| Parity ≥7 vs 1 | 3.70 (1.48–9.28)** | 5.04 (1.92–13.21)** | ✓ |
| **Child** |  |  |  |
| Age 36-59 vs 6-12 months | 0.00 (0.00–0.00)*** | 0.00 (0.00–0.00)*** | ✓ |
| *^1^*†One child randomly selected per mother (762 mothers with multiple children) | | | |
| Data are OR (95% CI). *p<0·05, **p<0·01, ***p<0·001. ✓=concordant significance, ✗=discordant. 54/56 coefficients (96·4%) showed consistent results. Only variables with p<0·10 in either analysis shown. Full results in appendix. | | | |
